# The Brain and Its Physiology

**Published:** 1846-10-01

**Authors:** 


I. The Brain and its Physiology; a Critical Disquisition on the Methods of determining the Relations subsisting between the Structure and Functions of the Encephalon. By *Daniel Noble*, Member of the Royal College of Surgeons of England. Octavo, pp. 450. London: John Churchill, 1846.

II. Examen de la Phrénologie. Par *P. Flourens*, Secrétaire perpetuel de l'Académie Royale des Sciences (Institut de France) &c. Paris, 1842. Pp. 115.

III. Quelques Considérations en Réponse a l'Examen de la Phrénologie de M. le Professeur *P. Flourens*. Par *M. S. de Wolkoff*. Baden, 1846.

It is scarcely necessary to observe that of all the departments belonging to the science of organization, that relating to cerebral anatomy and physiology is the most involved and obscure. Even setting aside all the unprofitable speculations as to the ultimate relations existing between mind and matter, an inquiry that embraces the mental faculties of men and animals; that regards the respective shares of the mind and of the external senses in the production of sensation; and that attempts to define the connexions existing between individual parts of the brain and individual powers of the mind, such an inquiry must be sufficiently difficult in its prosecution, and still more so in its successful solution. We do not therefore desire to imply, that there are any difficulties in this investigation offering an insuperable obstacle to the attainment of as complete a knowledge of the actions of the brain as of any other organ in the body; on the contrary, the progress made during the present century has not only added a vast number of individual facts to the pre-existing stock, but, which is infinitely more important, it has, by disclosing the clue to the true anatomy of the nervous system, laid the sure and certain foundation for the discovery of the whole of its physiology. This clue we hold to be the fact that the brain and spinal cord have a *fibrous structure*, a truth for which science is essentially indebted to Gall.

"The most accredited method," says Cuvier, in the Report to the Institute of France, speaking of the ordinary mode of dissecting the brain, "the most accredited method of the schools, is to take away successive slices of the organ, and to remark the appearances offered by each. This is the easiest in practice for the demonstration, but it is the most difficult for the imagination: the true relations of parts, which are always seen cut across, escape not the pupil alone, but the master himself." In allusion to the merits of Gall, M. Flourens, in his recent brochure, justly observes, "Gall was a great anatomist. The idea which he had of following and tracing the fibres of the brain is, for the anatomy of this organ, the fundamental idea."—*Examen de la Phrénologie*, p. 102.

It is true, indeed, that both of these writers attempt to deprive Gall of the renown attaching to the founder of cerebral anatomy; but a careful review of the whole question induces us deliberately to affirm, that to the German philosopher belongs the high merit of rescuing the anatomy of the brain from a state, in which it was impossible, by any talents or by any labour, to have discovered a single fundamental principle. In making this assertion, we are neither unmindful that the fibrous structure of the cerebrum was more or less known to Varolius, Leeuwenhoek, Vieussens, and others; nor that some of those anatomists insisted in their writings on the importance of tracing the relations of the several parts of the encephalon by following out the fibres. In the case of Vieussens particularly, although it is evident, from his having principally examined the brain by means of sections from above downwards, that he was not aware of the true method, it is remarkable that an anatomist who had so successfully traced the fibrous texture, as is indicated by several of his plates, especially the sixteenth, should neither have himself perceived the vast importance of his mode of procedure, nor yet have laid the true foundation for his immediate successors. Without inquiring how it came to pass that the interesting work of Vieussens (*Neurographia Universalis*) fell into the sort of discredit noticed by Cuvier, we would observe that fragments like those just pointed out, have almost invariably preceded in every branch of human investigation, the establishment of great principles; but they have never prevented posterity awarding to him, who, by his genius, his sagacity, and his industry, indelibly traces out a leading truth in science, the palm due to the discoverer, and so often withheld by envious cotemporaries.*

It may perhaps at first appear that the importance here assigned to the researches of Gall, is incompatible with the high claims of the late Sir C. Bell, as advocated in our article on the nervous system contained in a former number of this Journal—(*See Medico-Chir. Review, July* 1845.) Entirely abiding by the views previously announced, it would not, however, be difficult to show that, although our distinguished compatriot might have made out the respective offices of the roots of the spinal nerves, he could not have enunciated, or, at all events, have demonstrated the truth of those great laws which we regard as the fundamental basis of all neurology, had not the fibrous organization of the white substance been predetermined. How would it have been possible, for example, without such knowledge, to have established the all-important principles set forth in the following passages contained in the profoundly philosophic " Exposition of the Natural System of the Nerves of the Human Body?"

" The key to the system will be found in the simple proposition, that each

---

* It would be an injustice to pass by unnoticed the admirable researches of Reil into the fibrous structure of the brain; nothing can exceed the accuracy and clearness of his representations taken from dissections of the parts hardened by alcohol, and which have served as a guide for most subsequent investigations. Reil's Essays first appeared in the 8th and 9th volumes of the Archiv. für die Physiologie (1807-8), published by him and Autenreith; but it is essential to state that Reil mentions he commenced this series of inquiries as early as 1795, about the time when Gall, after a constant study of 20 years, delivered his first course of lectures at Vienna.

filament or track of nervous matter has its peculiar endowment, independently of the others which are bound up along with it; and that it continues to have the same endowment throughout its whole length. If we select a filament of a nerve, (for example, one of those in the compound nerve represented above,) and if its office be to convey sensation, that power shall belong to it in all its course wherever it can be traced: and wherever, in the whole course of that filament, whether it be in the foot, leg, thigh, spine, or brain, it may be bruised, or pricked, or injured in any way, sensation and not motion will result: and the perception arising from the impression will be referred to that part of the skin where the remote extremity of the filament is distributed." P. 11.

"If we were successfully to trace a nervous cord, (we shall suppose from a muscle of the fore-arm,) it would be found a simple filament, thread, or funiculus. We should then trace it into a compound nerve; perhaps the ulnar nerve; which we call compound, because there are in it filaments of motion and filaments of sensation bound together. At the root of the axillary nerve we should trace it into the composition of a fascis, where it forms the anterior root of a spinal nerve. Being further traced, it would merge in the anterior column of the spinal marrow; and traced into the base of the brain, it might be followed as a *tractus*, a streak of matter distinguishable from the surrounding substance, until it was seen to disperse and lose itself in the cineritious matter of the cerebrum. In all this extent, however combined or bound up, it constitutes one organ, and ministers to one function the direction of the activity of a muscle of the hand or finger." —*L. c.* p. 14, *Edition* 1824.

Reflecting on these splendid truths, and then turning to Gall's beautiful representations of the course of the ascending fibres from the pyramids of the oblong medulla, through the pons varolii, crura cerebri, thalamus, and striated body, to their radiation and passage into the cerebral convolutions, all becomes significant, precise, and clear. Does not the physiologist see at a glance in those invaluable plates the tracts by which, as through so many distinct paths, the mandates of the will are transmitted from the organ of volition, the cerebral hemispheres to the organs of motion, the muscles? Does he not follow, with as much facility as in regarding the wires of a galvanic trough, the conductors by which impressions made on the skin are carried to the organ of perception, the convolutions of the brain? Or, if the pathologist desires to know in what way it happens that a clot of blood effused in or near the optic thalamus or corpus striatum induces paralysis on the opposite side of the body, how clearly is that otherwise inexplicable phenomenon understood, by observing the decussation of the pyramids, and the radiating course of their fibres? We would even go further, and contend that some, and as it might seem, quite independent doctrines, if they have not sprung from the dissections of Gall, have at all events received from them some of the most satisfactory parts of the evidence in virtue of which they are assuming the character of fundamental truths. Of this number we conceive, are, for instance, the theories that the cerebral convolutions are the exclusive seat of intelligence —that the spinal system extends beyond the pons varolii—and that the gray, and not the white substance of the hemispheres, is the source of power.

We have been anxious on several accounts to place the anatomical researches of Gall in that pre-eminent point of view, to which we feel satisfied they are so well entitled. One of these reasons is that, as we shall be compelled in the course of the present article to dissent from the peculiar physiological doctrines of that distinguished man, we were anxious to show that no indisposition to receive information from their author had influenced our judgment. But another, and much more powerful motive, has been the conviction that the majority of physiologists do not appear to estimate at their real worth Gall's anatomical labours, nor even yet to perceive that all consistent and philosophical acquaintance with the cerebral actions, must repose on that method of dissecting the brain which he was the first firmly to establish. That these assertions are well founded would be made apparent by referring to the original report of Cuvier, and to the reception the new anatomy of the brain experienced for so long time in this country; and that it is still necessary to vindicate this as the only true system, is evident from the expresssion of a physiologist of deservedly high reputation, especially in reference to the brain, M. Flourens, to the effect that, "the anatomy of the memoir of Gall is only a very ordinary anatomy." (Examen, p. 54.) that there are several errors in Gall's anatomical doctrines we are most ready to admit; but the existing state of knowledge would easily have enabled M. Flourens to point out these without passing such a sweeping condemnation on the whole system, which is even inconsistent with the praise contained in the passage previously extracted.

We have in the foregoing remarks anticipated in some degree the principal object of the present article, which is to consider what are the means best adapted for the successful cultivation of cerebral physiology. This task has been undertaken by Mr. Noble in his "Critical Disquisition;" and we are bound to say he has displayed considerable literary research, much judicious observation, and a desire to elucidate the truth. A work of this character, being a review of the labours of other men, is not indeed likely to contain much novelty; we are not, however, on that account inclined to quarrel with the author, because such an inquiry, when conducted in an enlightened spirit, is well calculated to render good service in the cause of science. Mr. Noble, in his preface, justly condemns the application of exclusive methods of investigation; and has, in the following paragraph, shown the evils of such limited views with respect to the brain.

"Each division of natural inquiry is competent to yield its proper results; but when departmental students would deduce from any one branch of knowledge, conclusions that have no direct relation to it, error and confusion will be almost sure to characterise the issue of such attempts. When the vivisector anticipates, from his industry and dexterity, the determination of truths which do not sustain a disclosure by the method employed, he will not fail to be disappointed; and the same may be affirmed of the pathologist and the comparative anatomist. In the many efforts that have been made to shed light upon the functions of particular parts of the Encephalon, the fashion of the age, or the special pursuit, has generally settled the method of investigation; and so, in great measure, the custom continues to the present day. About thirty years ago, mutilations of living animals led to some decisive results in researches into the functions of nerves, and renown became justly attached to the experimenters; hereupon, vivisections for a time came into vogue, and every thing doubtful in physiology was to be tested by the fashionable mode. Later on, morbid anatomy became the subject of an all-absorbing interest, and thence was inferred an inexhaustible source of widely-extended discovery; how prematurely so, the world is beginning to see. The attention of some of our leading physiologists has of late years been more particularly directed to microscopic examination of the animal tissues, and to comparative anatomy; and these pursuits, as furnishing the means for the solution of all doubtful points in physiology, are, in many quarters, coming into transitory fame. All these sources of knowledge are important; they have each most useful ends or aims, beyond which, however, they cannot legitimately advance. Neither the extent to which they are studied, nor the talent and zeal with which they are investigated, can ever determine their adaptation to the discovery of truths to which they are essentially inadequate. The application of this argument to the particular instance of the Brain and its Physiology, constitutes the subject and purpose of the following pages." P. viii.

In his introductory chapter the author, after pointing out the deep interest and importance attaching to the functions of the brain, inquires how it is that the study of them has been so generally neglected by medical men. The most influential cause he conceives to be " the supposed hopelessness of any inquiry concerning the relations subsisting between mind and matter." In this view of the case we in some degree coincide, believing as we do that the whole question relating to psychical phenomena, as a branch of physiology, has come to be regarded as a profitless inquiry, owing to the fact that a large portion of the investigation has consisted of idle controversies upon the ultimate nature of mind, upon the essential connexion between the mind and the brain, and upon matters of a similar character. It is one of the best signs of the times in which we live, that the most distinguished men in every branch of science, have at length agreed, as by common consent, to investigate phenomena, to trace their relations, and to determine the laws on which they depend, thus obeying the practical impulse of the age, and leaving the dreamers in philosophy to struggle after the unattainable " ultimate facts" of Nature.

To this fundamental cause Mr. Noble would add errors of method; he neither approves of experiment, comparative anatomy, nor pathology as efficient means of investigation, taken singly or collectively; he " believes himself to be in a condition to show that each of these three methods, as leading to the primary fundamental evidence relative to the cerebral functions, is both defective and vicious; defective, by supplying insufficient data at the best; and vicious, by, in many instances, suggesting erroneous conclusions."—*L. c.* p. 13.

Having had some practical acquaintance with each of these modes of investigation, and moreover being convinced that they do in reality constitute, with the addition of a comprehensive knowledge of the human organization, the only means for unravelling this knotty question, we felt some curiosity to know what was the author's plan; and here it is:— " Structure associated with function—magnitude in the development of the former, in connexion with excessive manifestation of the latter—the size of certain parts of the brain, in alliance with corresponding powers of the mind—he believes to be the primary objects of inquiry."—(*L. c.* p. 14.) In other words, Mr. Noble is a thorough advocate for Phrenology, and occupies upwards of three hundred pages, or three-fourths of the entire volume, in illustrating and enforcing the doctrine of Gall. He has, however, given an interesting sketch of all the later researches of physiologists in general, with critiques on their value, and these chapters we proceed to notice.

The author commences his inquiry relating to vivisections with some remarks which embody an error so fundamental that we must in the outset protest against them. He says—

"The impossibility of gaining additions to our knowledge of the cerebral functions by mere dissection of structure, would appear to be generally conceded. No examination of the intimate constitution of the dead brain could ever do more than exhibit its physical qualities. The most powerful microscope could but reveal its molecular disposition. Nevertheless, a minute anatomical investigation of parts, the functions of which have already been determined, may shed considerable light on the mode in which these are performed; and the sympathies subsisting between one animal function and another, are sometimes beautifully illustrated, by demonstration of a correspondence in the organic arrangements. This, however, always presupposes that function is ascertained." P. 15.

That these are the views generally adopted by physiologists, we entirely doubt; and therefore protest, in the name of the English school, against the proposition, that the anatomical investigation of the brain does not form the basis of *all* inquiries into its functions; to make structure follow function would be totally to reverse the mode of investigation adopted by all the great discoverers in our science, from Harvey to Bell. The latter, in reference to a point immediately connected with this question, says, "my conceptions of this matter (the functions namely of the roots of the nerves) arose by inference from the anatomical structure, so that the few experiments which have been made, were directed only to the verification of the fundamental principles on which the system is founded." And so it ever must be—accurate anatomy, and a comprehensive knowledge of structure, preceding an investigation of function; this will continue to be, as it has hitherto been, the fixed order for arriving at sound physiological induction.

Nor are we much better satisfied with the estimate placed by the author on experiments practised on living animals. That these have been multiplied most unnecessarily, and therefore unjustifiably; that they have been frequently misinterpreted; and that consequently they have often led to conflicting and erroneous inferences, all this we are as willing to admit as Mr. Noble himself; and if he had confined his criticisms to the instance of the brain, there would have been less ground for dissent. But when the great truth established by the celebrated experiments on the roots of the spinal nerves is, for the purpose of throwing discredit on vivisections, spoken of hypothetically (*l. c.* p. 31), we must again protest, and inquire if, from among the crowd of writers on the phrenological doctrine, including the masters themselves, Gall and Spurzheim, a single fact can be brought forth from the inferences, assumptions, and probabilities, making up the main part of their evidence, which will for a single instant bear comparison with the fundamental principles that have resulted from Bell's discovery. Even the apparent inconsistencies which the ready tongue of envy attempted to proclaim as proofs of error, such as that irritation of the posterior or sentient roots induced under certain conditions, contractions in the muscles, were only completely explained by means of the vivisections connected with the next great advance in neurology, the discovery, by Dr. Marshall Hall, of the excito-motory power, itself essentially due to experiments akin to those here so much depreciated.

In the Chapter "On Comparative Anatomy as the Primary Aid to discovery of the Cerebral Functions," there are some judicious observations well worthy of attention. Among these are the remarks illustrative of the important law now generally recognised that, *size or amount of nervous tissue, constitutes a direct element of functional power*—a law which, as the author observes, brings the brain and nervous system into harmony with all other created things.

"In physics, the law is obvious at once. Size of any given body is always regarded as not the least important property, in an estimate of its power; and, in the vegetable world, productive energy is always qualified by size or amount of structure. In the animal kingdom, independently of the nervous system, the influence of organic size upon energy of function is everywhere admitted. Capacious lungs are more likely to be vigorous in the execution of their office than smaller ones. A large heart will usually propel the circulating fluid with more energy than one that is small; and a like principle holds good with respect to the abdominal viscera; in short, observation has revealed the truth, that the law which regulates the vigour and energy of inorganic matter, of vegetable and general animal life, prevails also in the particular constitution of the brain and nerves. Thus, as a general rule, the sentient nerves bear a proportion in size to the degree in which the power of sensation is possessed. Desmoulins states that, in man, the posterior roots of the spinal nerves, being for sensation, in supplying the superior extremity, have at once an excess of volume and of surface, at least five times greater, both for each individual fibre, and for the bundle resulting from them, than the anterior roots which are appropriated to motion. And this fact corresponds with the exquisite sense of touch possessed by the human hand. The same author further mentions, that the single nerve of feeling ramified on the tactile extremity of the proboscis of an elephant, exceeds in size the united volume of all the muscular nerves of that organ." P. 38.

It is evident that, to render comparative anatomy of service in determining the respective offices of the different cerebral masses in man, it is indispensably necessary to ascertain what particular parts correspond to each other in the several classes of vertebrate animals. But this pre-requisite is admittedly one of the most difficult points in comparative neurology. "Thus, with respect to the first, or anterior pair of knots of nervous substance, found within the heads of fishes, they are considered by Arasky, Serres, Desmoulins, Carus, and Tiedemann, to be analogous to the cerebral hemispheres in man; whilst Collins, Monro, Camper, Ebel, Treviranus, and Cuvier, regard them merely as connexions of the olfactory nerves. Again, the second cerebral mass is believed, by the last-named physiologists, to constitute the cerebral hemispheres; the former group consider it to form the analogue of the *corpora quadrigemina.*" Then, again, if even these points were settled, there remains a greater difficulty, a knowledge of the precise actions, habits, and instincts of the lower animals, or what the author terms, by a new application of the word, their *psychology*.

We shall not follow the author in his somewhat tedious review of the various hypotheses, resting on comparative anatomy, which have been put forth in late years as to the respective seats of the mental powers, because it is certain that at present there is not sufficient evidence to disclose what will in reality constitute the last things to be known in cerebral physiology. One exception may be allowed in reference to the Cranioscopy of Carus, as it has attracted some notice among physiologists and is probably unknown to many of our readers. The theory is that the encephalon is divisible, both as to function and structure, into three masses, the basis of the arrangement being obtained from the brain of the fish; the anterior consisting of the true cerebral hemispheres, the middle of the corpora quadrigemina, and the posterior of the cerebellum.

"In man, the characteristic feature is the enormous development of the hemispheres. Farther, I have shown that these three cerebral masses, which appear almost in the same relations in the early human embryo as in fishes (that is to say, the middle central mass is the largest), are always to be recognised as endowed with a peculiar function. The posterior cerebral mass is the centre of the primitive fibres of the muscular nerves, and of those of sex. In the middle cerebral portion, the primitive fibres of the reparative organs are collected; while in the anterior cerebral mass essentially, we find the primitive fibres of the organs of sense, through the medium of which we derive our ideas of sensible objects, and in a higher degree our knowledge. In short, the three cerebral masses stand in relation to the following psychological qualities:—

"1. The anterior cerebral mass (or the hemispheres) is related to the power of representing ideas, to that of recognising and distinguishing them, and to that of imagination.

"2. The middle cerebral mass (corpora quadrigemina) is related to the feeling of the condition of our own organic life (common sensation); and to sentiment, or to the feelings which result from the combined action of all our moral faculties.

"3. The posterior cerebral mass (cerebellum) is related to will, desire, and the instinct of generation.

"As the fundamental elements of mental life are only three—to know, to feel, and to will—so are these three masses the essential portions of the cerebral structure. From these three proceed the three important nerves of sense, those of smell, vision, and hearing, which again correspond to three great regions of the cranial structure, the forehead, the middle head, and the hinder head."—*Critical Disquisition*, p. 55.

The evidence derived from pathology we regard as one of the primary sources of information elucidative of function; not so Mr. Noble, who affirms that these researches, however much they may corroborate truths otherwise obtained, "are, in their very nature, unsuitable as *direct* guides in the prosecution of Cerebral Physiology." So far from this being the fact, some of the most essential, most certain, and, for future progress, most promising facts, *have been proved*, whatever might have been from other investigations surmised, by carefully comparing symptoms during life, with the lesions discovered after death. The general results thus gained are briefly but well stated by Dr. Brigham, an American writer, in the following passage, quoted by Mr. Noble: "First, we have, it appears to me, ascertained from pathological observations, that the functions of the cineritious and the medullary portions of the brain are quite different; that the cineritious portion of the brain is more particularly concerned in intellectual operations, while the office of the medullary part is to conduct sensation and volition; that when the medullary part is alone affected, disturbance of motion ensues, but not of the intellect; and that when the cineritious portion is diseased, the intellect is alone affected."—(*L. c.* p. 85.) Too much importance is, perhaps, here attached to pathology with respect to the light thrown by it on the distinctive actions of the gray and fibrous structures; but every day's experience corroborates the justness of the other statements. Do we not find that, when the gray convolutions are in any way implicated, as by the pressure of a clot of blood, by an abscess, or by inflammation of the membranes, invariably the intellect is affected, that there is either coma or delirium; whilst in effusion of blood into the substance of the organ, or about the optic thalamus and corpus striatum, and in deep-seated inflammations of a local character, is it not the fact, to borrow the language of Dr. Stokes, that "the most important symptoms are those which are derived from the sympathetic affections of the muscular system," and to which we would add, also, from special derangements of sensation? We speak here of well-marked cases and of permanent results. It is doubtless true that the motor power is often affected in irritation of the surface of the brain, as from spicula of bone in the dura mater inducing convulsion; and also that effusion into the substance may affect the intellect; but the best pathologists are agreed that these phenomena arise from a combination of morbid changes, of which the most essential is a disturbed state of the vascular system within the head. It is the frequency of such general implications which has caused so much difficulty in drawing exact conclusions; but the improved acquaintance with the laws of nervous action that has resulted from the inquiries of the last 30 years, will ultimately enable the physiologist to separate the incidental from the essential, and thus to fix with precision the offices of the various masses of the encephalon.*

---

* It is not an unusual thing to hear complaints of the unsatisfactory character of cerebral pathology from others besides the author; not a few practitioners holding all evidence derived from this source, when minuteness and therefore accuracy is attempted, as being, to say the least of it, doubtful or speculative. It is so essential to a more sound knowledge in this interesting class of disease, that confidence should be placed in the morbid anatomy of the brain, that we are induced to insert the following instructive case, related by M. Mahot (Archiv. Gen. de Medicine, 1845). A soldier aged 22 years, was found insensible on Jan. 8th, foaming at the mouth, and with the *left* arm and leg rigidly contracted. Having been restored by dashing cold water on the face, he had a second fit in four hours afterwards. Extreme weakness of the left arm and leg with imperfect motion continued for ten days. Subsequent to an attack of measles, paralysis as to motion, but not of sensation, was observed, on the *left* side of the face, arm, and leg: the intellectual faculties perfect. Pulse 50 to 55. On the 24th of January a fit of insensibility with stertorous breathing; and on the 26th the patient could not turn the *right* eye outwards. Repeated attacks of vomiting and convulsion supervened and continued till April 17th, when the patient died. The post mortem disclosed extensive effusion, &c. but the most interesting fact was the existence of a firm tumour, of the size of a chesnut, seated on the *right* side of the Pons Varolii, close to the median line, pressing on the origin of the 6th or external motor nerve of the eye. With the aid of a comprehensive knowledge of the organization of the cerebro-spinal centre the interpretation of the complex symptoms of this case becomes plain and simple:—the rigid contraction of the arm and leg in the first instance, arose from the irritation of the motor apparatus of the top of the spinal cord by the tumour, which at this period did not produce compression; the paralysis of the left arm and leg resulted from the pressure on the right side of the median plane of those motorial fibres, which subsequently, in the decussation of the anterior pyramids, get on the left side of the body: the paralysis of the abductor oculi on the right side depended on the

We now approach that which the author, with other phrenologists, believes to be the *true system of cerebral physiology.*

" Development of the brain constantly influences the manifestations of mind; if the quantity of cerebral structure in particular regions be considerable, some related power or quality of the conscious principle will, under ordinary circumstances, be displayed in unwonted energy; and, with great deficiency in volume of brain in the same region, the corresponding power or quality will in every case be but feebly manifested; and not only so, but changes wrought in one set of conditions will often be recognisably induced in the other. Under all these circumstances, the idea of causation becomes irresistible; the induction, that the particular power is organically connected with the particular structure, is philosophically gained."—*L. c.* p. 97.

The evidence of physiology in general is affirmed to support this doctrine, " large size and perfection of structure, associated with great vigour and vivacity in function, modifications in the one corresponding with those in the other—the observation of these things has led to the existence of physiology as a science." But here commences the contrariety of opinion between, we may say, physiologists on one side and the phrenologists on the other; the position last quoted is granted by both parties, but how is it to be interpreted as regards the brain? For it may be said the cerebrum is one organ, the seat of consciousness, intellect, mind, or whatever term may be used to signify the intellectual faculties collectively, and that it enlarges in the ratio of mentality; and this view is as compatible with the principle in question as with that which assigns to the brain a multiplicity of organs, each being the independent seat of a special faculty.

As the ultimate fate of Phrenology cannot, it is certain, be resolved by the method more ordinarily pursued, at least in this country, that namely of comparing the configuration of the head and the relative development of the various convolutions, with the mental habits and aptitudes of the individuals examined, it is desirable to return to the more philosophic procedure of the founder of the system, and to inquire how far it is in harmony with the general laws of physiology. " Gall," says M. Flourens, " argues from the independence of the external senses to the independence of the faculties of the soul; he reasons upon an apparent analogy, which in reality conceals a profound dissimilitude: this is a capital and general error."—(*L. c.* p. 83.) The pupil, and to a certain extent collaborateur, of Gall, Spurzheim, reasons in the same manner: " in the nervous system, says he, we find the five external senses, separate from and independent of, each other; their functions are attached to different organs, and so they can exist separately: it is the same with

---

compression of the sixth nerve, situated immediately below the tumour: the disturbance of the breathing and the vomiting proceeded from the irritation of the great centre of those excito-motory actions, the medulla oblongata, and to the same cause the convulsions were owing: the intellect was unaffected, because all the permanent mischief was seated below the organ of the mind, the cerebral hemispheres, and these consequently were only occasionally involved from some incidental and transient cause, most probably deranged circulation. The only symptom involved in any obscurity, is the paralysis of the motor root of the left fifth pair: but the precise origin of this is not known, though no physiologist will doubt that it is such as would, if determined, explain the facial palsy.

the internal senses, and we maintain that there is a special organ for each species of feelings and thoughts, as for each kind of external sensation." As the distinguished Secretary of the Institute and now Pair de France, does not show in what the error he denounces consists, we shall take the liberty of supplying, as far as our humble ability will permit, the omission. And we are the more induced to do this, because among thoughtful inquirers, one of the strongest grounds for regarding phrenology favourably, has been the belief that the brain was investigated by Gall in strict accordance with the principles of physiology; and that, in attributing a plurality of independent organs to that body, he was adopting precisely the same plan as that recognised by all authorities in respect to the rest of the body.

If, in the above comparison, any inference is attempted to be drawn from the existence of distinct organs of sense, apart from their associated nerves, and this we imagine is the way in which the question has been usually regarded by the advocates of phrenology, a little consideration will show that the things attempted to be compared are totally dissimilar. The existence of distinct sensual apparatus, is a provision made necessary by the different qualities of matter: the apparatus of the eye is essential to the refraction of light, that of the ear to receive and transmit the vibrations of sound, and so on. If this be the comparison signified, what it may be asked can there be in common between mere physical instruments, for such are the mechanical parts of the organs of sense, and the subtle centre of animal life? If, however, it be the *nerves* of sense which are intended, we admit that each of them has its own peculiar endowment, like the nerves of motion and of common feeling, one being capable of transmitting the impression of light, another that of sound; but here again a necessity exists, for it is apparent the several nerves of special sensation, must be diversely endowed to transmit the impression of different physical agents. To have a true basis for the inference attempted, the phrenologists ought to be able to prove, not merely that there are, in obedience to the laws of innervation, differently endowed conductors leading from the organs of sense, but also *different organs of perception,* one for light, another for sound, &c. Now this evidence is not only entirely wanting, but the general conviction among the successful cultivators of physiology is, that there is only one central organ of perception, or sensorium commune, a conviction resting on observation and experiment. It is justly observed by M. Flourens, that when portions of the hemispheres are removed in living animals, no matter from what region, whether in front, behind, above, or on the sides, instead of any one particular faculty being thereby destroyed, the general intelligence is at first weakened, and afterwards, when the ablation passes certain limits, extinguished; or, in other words, the operator does not find, which ought to be the case if Gall's hypothesis were correct, first a loss of sight, then of hearing, then of feeling; but what he does observe is that "so soon as one sensation is lost, all are lost; that so soon as one faculty disappears, all disappear."—*Examen,* p. 23.

The result of this, which may be termed the philosophic mode of treating the question, is then that Gall has compared together things which are essentially different—the brain, the instrument of power, and the nerves, the mere conductors of impressions: as well might it be said that, because there are in the galvanic telegraph special conducting wires to the several stations, there must also be a distinct battery for each.

Another of those general questions involved in the reception or refutation of Phrenology, is that relating to the nature of the mental powers. "The psychologist," says Dr. Todd, "must determine what are, and what are not, fundamental faculties of the mind, before the physiologist can venture to assign to each its local habitation." Now, although we are willing to grant that the principle which assigns special organs to special faculties may be true, whilst the precise number of the faculties and the exact situation of the organs themselves may be still sub judice; yet, when we come to weigh the evidence pro and con, the objection just quoted on the one hand, and the analysis of the mind advocated by the new school on the other, must enter as important elements into the inquiry. The first thing which challenges scrutiny is the minute subdivision of the primitive powers. Some of these are so closely allied that no unbiassed person would hesitate to attribute them to the same mental quality; whilst others are so insignificant, such as "weight and resistance," that the wonder is how they ever could have found a place among the fundamental "facultés intellectuelles." It is not surprising when refinements so subtle as these are essayed, that there should be discrepancies, even between the leaders of the sect; that, whilst Gall should admit 28, Spurzheim, by only adding eight new fundamental faculties, should raise the number to 35!

There is, however, much more profound disagreement than this; for Gall, not content with his 28 primitive powers, attributes to each individual faculty—perception, memory, judgment, imagination, will, &c., so that in fact each faculty becomes an individual intelligence complete in itself. Spurzheim dissents from these views, affirming that the facts adduced "do not prove the conclusion." We would go further and inquire with Flourens, "with all these individual intelligences, what is the general intelligence properly so called? It might be, just as one pleases, either an attribute of each faculty, or the collective expression of all the faculties, or even the simple result of their common and simultaneous action: in a word, it would be no longer this faculty, positive and unique, which we understand, which we conceive, and which we all feel within ourselves, when we pronounce the word, soul or intelligence.—*L. c.* p. 25.*

---

* It may be thought that the discrepancies noticed above were dependent on the infancy of the science, or even upon the well-known controversy existing between Gall and Spurzheim. We find, however, the same kind of discordant statements still prevailing: thus M. Vimont, "phrenologiste très decidé et anatomiste très habile," according to M. Flourens, and highly lauded by Mr. Noble, affirms that "the work of Gall is more adapted to induce error than to give a just idea of the seat of the organs;" whilst, with reference to Spurzheim, it is contended that he has placed one organ on the frontal sinus, and another upon the muscles inserted in the occiput. It is somewhat remarkable after such critiques that M. Vimont should have inscribed on the cranium of a goose the names of no less than 29 faculties; among which we find, "extent, distance, geometrical sense, resistance, order, time, language, eventuality, construction, musical talent, imitation, comparison, sweetness"! All we can say is, that this must have been indeed a marvellously clever goose, and a worthy rival of the learned pig.

If the general arrangement of Gall be a difficulty to the uninitiated, assuredly his more minute analysis of the inter-dependencies and correlations of the several mental faculties, will not be more satisfactory. Who, for instance, would have thought of seeking in the propinquity or remoteness of the organs of colour, form, and imagination, for an explanation of those mental aptitudes which make or mar the painter? And yet we are told that, "as the organ of the arts is placed far from the organ of the sense of colour, this circumstance explains why the painters of history have been rarely colourists:" this is in truth mapping out the mind and enchaining the ethereal spirit.

Having already devoted so much space to this subject, we can only select one or two of the individual faculties, as examples of the kind of evidence current in the phrenological school. It was the opinion of Gall that the two organs of which the localization was the most surely established, were the cerebellum, the seat of the instinct of propagation, and the posterior cerebral lobes, the organ of the instinct of progenitiveness. In this view Mr. Noble coincides, for he states he "is one of those who think with Georget, that the organic connexion of the sexual instinct with the cerebellum is probably that particular point of the phrenological doctrine in favour of which the largest amount of proof is obtained"—*L. c.*, p. 252. In support of this opinion several cases are adduced, which have already been published either by Gall himself or by Dr. Combe and others. In two of these impotence was induced in consequence of blows from fire-arms, which had grazed the nape of the neck; in another case a ball, which had traversed the muscles of the neck, grazed the inferior occipital swellings, and atrophy of the testes and penis occurred for a time, though in two months all the symptoms disappeared. Two cases are also quoted from Dr. Stokes, in which it was supposed by that able physician that a connexion was exhibited between disease of the cerebellum and visible excitement of the external organs.

Of the major portion of this evidence all we can say is, that no unbiassed physiologist would receive it in proof of the function assigned to the cerebellum. In the three first cases related by Gall, there was no evidence of that organ being specially or even at all implicated: in the first of Dr. Stokes's cases there was not only effusion of blood into the cerebellum, but also in the hemisphere of the cerebrum; and, moreover, paraplegia existed, showing mischief in the spinal cord, to which the priapism ought doubtless to be attributed. In the injuries inflicted on the nape of the neck, it might at all events, with equal or greater probability be inferred, that the peculiar affection of the genital organs was the result of the upper part of the spinal cord being irritated. Many facts might be adduced in support of this view of the case: thus in injury of the spine producing paraplegia, priapism is a most frequent occurrence; and the emissio seminis that takes place in hanging has been attributed, we think justly, by the most sound physiologists to spinal irritation. But more than this there is positive proof both of the sexual desire and power existing where the cerebellum could not by possibility exert any influence. In the first place, there is the well-known case related by Andral of a half idiotic girl, 12 years old, who showed a precocious tendency to the passions of her sex, was addicted to masturbation, and in whom it was found after death that the uterus, fallopian tubes, and ovaries were fully developed, and yet the cerebellum was totally absent. Then, again, Brachet details an instance in which a man perfectly paraplegic became a father; to which may be added the more questionable case recorded in history of Albert the second, Duke of Austria, who was attacked with a paralytic disorder which deprived him of the use of the lower extremities, but who at a time when his increasing disorders seemed to preclude all prospect of issue, became the continuator of his race and the father of four sons.

When so much difficulty is experienced in assigning the special actions of the cerebellum, a part marked out by recognisable limits, for it should be remembered that, although the phrenologists have settled the matter to their satisfaction, the great majority of physiologists more or less agree with Flourens, that it is connected with locomotion—it is not surprising that, in proceeding to the convolutions of the brain, the obstacles in the way of localising their actions are infinitely increased. An objection which must with all unprejudiced persons have great weight is the fact often urged, that there is no natural limitation of the convolutions corresponding with the assumed special cerebral organs; on the contrary, the anfractuosities run continuously, and often to a great extent, in the various regions of the hemispheres; so that, as may be seen by contrasting the brain with the various plates of Gall and Spurzheim, figures, indicating several distinct "organs," are placed on different parts of the same continuous convolution. Mr. Noble contends that these objections arise from an "utter ignorance of Nature's plan in the development of the nervous system, *which, as a rule, exhibits no mechanical divisions coincidently with distinctness in function*"—(*L. c.*, p. 237); and various instances are adduced in corroboration of this position. These supposed proofs, we are compelled to say, are not judiciously chosen, and most of them are moreover erroneous. The first assertion is, that "the trunks of the nerves are composed of filaments in subservience to various functions, and that the several filaments are absolutely undistinguishable in their relation to function." With the glaring instance of the two distinct roots of the compound spinal nerves before our eyes, this, to say the least of it, is a bold assumption; but we go farther, and affirm that cautious microscopists can detect physical differences both between the nerves of special sense, as the optic and the auditory, and the nerves of motion, and even between these last and the nerves of common sensation. The second assertion is, that there is no reason to believe that the distinct columns marked out by the longitudinal furrows on the spinal cord and medulla oblongata have distinct offices; but, in opposition to this, it would not be difficult to show that the best physiologists think the above fasciculi do minister to separate functions, although these at present are not determined, and in this opinion we entirely coincide. The last reason embodies, quite unintentionally we are satisfied, an anatomical error; the author, in noticing the opinion entertained by many investigators, that the glosso-pharyngeal nerve has more offices than one, adds that this inference is deduced from the supposed manifestations, and not from any mechanical division. Now that which to our mind has always been the most satisfactory proof of the motor as well as sentient office of the nerve in question is the fact, that it precisely resembles the compound spinal nerves, having two distinct roots with a ganglion (*g. jugulare,* of Müller) on its posterior fasciculus.

A careful consideration of the nervous structure has brought to our mind the fixed conviction, that diversity of function is invariably accompanied by diversity of structure; and this being the case, the question recurs whether the apparent identity of structure and fusion of substance exhibited in the several convolutions of the brain, these being extensively continuous, is compatible with the assigned distinctness of function. We do not wish to affirm that there may not exist intimate and yet essential structural modifications in those bodies undiscoverable at present; all we desire to show is, that up to this time no such organic difference has been detected, and especially that the position taken up by the author is untenable. In reference to the locality of the convolutions there is a difficulty of which we have seen no satisfactory explanation: what is the office, the rôle being already complete without them, of those numerous convolutions placed at the base of the brain, of the insula for example, and on the flat sides of the hemispheres, of which no sufficient account is given, unless, like waifs claimed by the lord of the manor, they are to be thrown to their nearest neighbours? We cannot pursue this branch further, and therefore we must refer our readers to the chapters in which the author endeavours to prove the harmony of Gall's physiology with structural and comparative anatomy.

One of the most important points connected with Phrenology is the great light it is believed by many eminent physicians to throw on the causes, management, and results of mental diseases; and we at once admit that the opinions of these gentlemen are deserving of the most respectful attention. The author, who has two chapters on this subject, is most hopeful of the advantages to be derived from his favorite doctrine.

"Gall's method of observation having proved—what previously was but a well-grounded hypothesis—that the brain is the organ of the mind, it follows that a morbid condition of this structure should tend to the depraved manifestation of the mental phenomena. Phrenology, moreover, having shown that the encephalic mass is a congeries of organs—the office of each being to display an individual faculty of the mind—has afforded a flood of light to illumine the once obscure path pursued in the investigation of mental derangement; and, indeed, it bids fair, if but once generally understood and assiduously cultivated, to dissipate much of the intricacy and mystery that formerly involved this subject of inquiry—for so many ages the opprobrium, not only of medical men, but of the civilised world at large."

"By demonstrating that diseased manifestation of the mental functions is associated with corresponding derangement of the material organ; by affording a reasonable and practical, if not perfectly accurate, analysis of the human mind, in an enumeration of most of its primitive powers and inclinations; by exhibiting the organic connexion between special parts of the brain and determinate mental faculties; by showing that the phenomena of insanity must be regarded as pathological conditions of the brain—phrenology (employing this term in its widest acceptation) offers for the first time a distinct clue to a system of cerebral pathology and cerebral medicine, as experimental in its nature, and as rational, as that which is afforded by other branches of the healing art." *L. c.* p. 336.

The history, especially of monomania is supposed to receive great elucidation from Gall's physiology; for as there are so many independent organs, one of these may be morbidly disturbed, and with it the associated function, leaving the rest of the cerebral organization and actions anaffected. This is doubtless a plausible mode of accounting for the well-known peculiarities of monomania; but so to some persons was the explanation offered by Dr. Wigan of certain forms of mental aberration; and indeed there is nothing in the supposition of two cerebra and a dual mind existing in one individual, more opposed to the common views of mankind, than the doctrine of Gall concerning multiple brains and distinct intelligences. These considerations induce us to request our readers to contrast the past progress of phrenology, with the principles in accordance to which it is now agreed science ought to be cultivated; and to remember that, in the investigation of abstruse natural phenomena, something more than plausibilities are required. Great truths have been invariably established by a few simple and unquestionable facts; so much indeed is this the case that it is justly regarded as a suspicious circumstance, when any theory requires of its advocates a crowd of probabilities and inferences for its support. Now it is precisely in these two respects that phrenology fails in realising the tests of truth; it has been overlaid with an endless amount of arguments, inductions, and deductions, and is supported scarcely by a fact that is not either questionable or questioned. And yet, considering how long and how eagerly the subject has been prosecuted, the time is come when more positive proofs ought to exist.

Even as regards one of the most promising sources of evidence, morbid anatomy, the results hitherto afforded have been insignificant; and we entirely coincide with those authorities who, to borrow the words of Mr. Noble, have objected, though as he contends unjustly, "that the morbid anatomy of insanity does not furnish that corroborative evidence of the soundness of Gall's physiology of the brain which general physiology receives from the same source." Considering the multiform varieties of insanity—the marked cases so constantly recurring, in which the disturbance is determinately restricted to some peculiar hallucination, all else being sound—the manifold instances in which for a long series of years, certain faculties are permanently deranged, having, it is affirmed, cerebral organs widely separated from each other, and in which, consequently, morbid changes of structure should, by contrast with the healthy surrounding parts, be strikingly apparent; considering all these things and the slight indications of corresponding, that is local, morbid changes detected, the conclusion forced upon the mind is, that the actual evidence falls altogether short of those precise allegations in which phrenology abounds. How often, for example, is it found that where special faculties only have been affected, the morbid changes in the brain and its membranes are general, instead of being local and restricted; this general organic disturbance is, in fact, by far the most common alteration detected, whatever may have been the peculiar form of insanity. It is often asserted in answer to all these objections, that "aberration of function is not *always* succeeded by appreciable change of structure." But we might appeal to pathologists and ask, if, in chronic cases of mental derangement this is not the exception; the rule being, that to the practised eye morbid changes, usually as we have said more or less general, such as opacity and thickening of the arachnoid, effusion of serum, alteration of the gray and fibrous substance,

&c., do not present themselves. The fact is then, that in chronic insanity there is usually the evidence of pathology, but it indicates a general and not a local disease of the encephalon ; the proof exists, but it is not of the right kind.

The author observes, " it is a very interesting circumstance to notice, that *all* who have to deal practically and professionally with mind, and who at the same time understand phrenology, recognise the light which it sheds on their path."—*L. c.* p. 404. Testimonies from various individuals are then adduced in support of this position from one of Dr. Combe's work. That much benefit has resulted from the systematic study of the mental powers, which has attended the discussions of Gall's theory, and especially from the great attention that has been paid to the broad distinctions so much insisted on as existing between the affective and intellectual faculties, we are quite prepared to grant; and herein the phrenological school has conferred great and lasting benefits on society. But beyond this, we believe little has been gained; at all events we must enter our protest against the author's assertion, that *all* persons engaged in mental training have derived the advantages from phrenology indicated in the above passage.

In the course of this article several allusions have been made to the pamphlet of M. Flourens. We have only to add, that it contains many judicious observations all worthy of consideration ; but we agree with M. De Wolkoff, that it is most reprehensible to mix up, as M. Flourens has done, religious doctrines with mere matters of science. As to the " *Reponse*" of M. De Wolkoff, it contains little worthy of remark ; this author, however, although he informs us he had great joy, after a painful study of the metaphysicians, when the work of Gall fell into his hands, believing he had at last found the treasure for which he was so long seeking, does not, after all, seem very much satisfied with having, for these twenty years past installed in his library the works of the phrenologists in the place previously devoted to those of the metaphysicians. The following passage would rather indicate that he has yet to seek for that ultimate rest, all engaged in philosophic speculations so earnestly desiderate, a fixed conviction : " in discovering in the human brain a constant disposition of the convolutions, it is not surprising that the phrenologists thought they might well be the seat of the diverse mental faculties. But beyond this it is only a supposition, and, however probable this supposition may appear to be, it would be wrong to regard it as an indubitable truth."—*Quelques Considérations au Réponse*, &c. p. 8.

Before concluding our remarks, it is due to Mr. Noble to state, that although we have, in the discharge of our impartial duty, felt it to be necessary to oppose the physiological views he has adopted, we are most ready to bear our testimony to the talent and extensive information he has evinced ; and we cannot for this reason but regret that he had not selected a more promising field for their exercise.

